# Programmatic Management of Drug-Resistant Tuberculosis: An Updated Research Agenda

**DOI:** 10.1371/journal.pone.0155968

**Published:** 2016-05-25

**Authors:** Carole D. Mitnick, Carly A. Rodriguez, Marita L. Hatton, Grania Brigden, Frank Cobelens, Martin P. Grobusch, Robert Horsburgh, Christoph Lange, Christian Lienhardt, Eyal Oren, Laura J. Podewils, Barbara Seaworth, Susan van den Hof, Charles L. Daley, Agnes C. Gebhard, Fraser Wares, RESIST-TB (Research Excellence to Stop TB Resistance) and GDI (Global Drug Resistant TB Initiative)

**Affiliations:** 1 Department of Global Health and Social Medicine, Harvard Medical School, Boston, MA, United States of America; 2 Partners In Health, Boston, MA, United States of America; 3 Boston University School of Public Health, Boston, MA, United States of America; 4 Access Campaign, Médecins Sans Frontières, Geneva, Switzerland; 5 KNCV Tuberculosis Foundation, The Hague, Netherlands; 6 Amsterdam Institute for Global Health and Development, Academic Medical Center, University of Amsterdam, Amsterdam, Netherlands; 7 Center of Tropical Medicine and Travel Medicine, Department of Infectious Diseases, Division of Internal Medicine, Academic Medical Center, University of Amsterdam, Amsterdam, Netherlands; 8 Division of Clinical Infectious Diseases and German Center for Infection Research Tuberculosis Unit, Research Center Borstel, Borstel, Germany; 9 Department of Medicine, University of Namibia School of Medicine, Windhoek, Namibia; 10 Department of Medicine, Karolinska Institute, Stockholm, Sweden; 11 Global Tuberculosis Program, World Health Organization, Geneva, Switzerland; 12 Department of Epidemiology & Biostatistics, Mel and Enid Zuckerman College of Public Health, Tucson, AZ, United States of America; 13 Division of Global HIV/AIDS and Tuberculosis, US Centers for Disease Control and Prevention, Atlanta, GA, United States of America; 14 Heartland National TB Center, Texas Center for Infectious Diseases, San Antonio, TX, United States of America; 15 Central Office, KNCV Tuberculosis Foundation, The Hague, Netherlands; 16 Department of Medicine, National Jewish Health and the University of Colorado Denver, Denver, CO, United States of America; 17 Technical Division, KNCV Tuberculosis Foundation, The Hague, The Netherlands; Indian Institute of Science, INDIA

## Abstract

**Introduction:**

There are numerous challenges in delivering appropriate treatment for multidrug-resistant tuberculosis (MDR-TB) and the evidence base to guide those practices remains limited. We present the third updated Research Agenda for the programmatic management of drug-resistant TB (PMDT), assembled through a literature review and survey.

**Methods:**

Publications citing the 2008 research agenda and normative documents were reviewed for evidence gaps. Gaps were formulated into questions and grouped as in the 2008 research agenda: Laboratory Support, Treatment Strategy, Programmatically Relevant Research, Epidemiology, and Management of Contacts. A survey was distributed through snowball sampling to identify research priorities. Respondent priority rankings were scored and summarized by mean. Sensitivity analyses explored weighting and handling of missing rankings.

**Results:**

Thirty normative documents and publications were reviewed for stated research needs; these were collapsed into 56 research questions across 5 categories. Of more than 500 survey recipients, 133 ranked priorities within at least one category. Priorities within categories included new diagnostics and their effect on improving treatment outcomes, improved diagnosis of paucibacillary and extra pulmonary TB, and development of shorter, effective regimens. Interruption of nosocomial transmission and treatment for latent TB infection in contacts of known MDR−TB patients were also top priorities in their respective categories. Results were internally consistent and robust.

**Discussion:**

Priorities retained from the 2008 research agenda include shorter MDR-TB regimens and averting transmission. Limitations of recent advances were implied in the continued quest for: shorter regimens containing new drugs, rapid diagnostics that improve treatment outcomes, and improved methods of estimating burden without representative data.

**Conclusion:**

There is continuity around the priorities for research in PMDT. Coordinated efforts to address questions regarding shorter treatment regimens, knowledge of disease burden without representative data, and treatment for LTBI in contacts of known DR-TB patients are essential to stem the epidemic of TB, including DR-TB.

## Introduction

Drug-resistant tuberculosis (DR-TB) threatens global TB control and is a major public health concern. The World Health Organization (WHO) estimates that 480,000 new cases of multidrug-resistant tuberculosis (MDR-TB), tuberculosis resistant to at least isoniazid and rifampicin, occurred in 2014.[1] Of these, an estimated 8.7% had extensively drug-resistant tuberculosis (XDR-TB), defined as MDR- TB with additional drug resistance to at least one fluoroquinolone and a second-line injectable drug. Effective management of drug-resistant tuberculosis (DR-TB) requires prevention, case detection, care and treatment, surveillance, drug management, and monitoring and evaluation of program performance. These activities should be coordinated by national TB control programs, and are referred to collectively as the "programmatic management of drug-resistant tuberculosis" (PMDT).

Guidance from WHO on PMDT has been available since 2006, and updated guidance based on new research, technology, and expert opinion has become available over the past decade.[2–5] Unfortunately, progress on the scale up of PMDT has been decidedly slow. Despite an increase in the number of persons with MDR-TB detected, from 46,897 in 2009 to 122,618 in 2014, approximately 75% of TB patients estimated to have MDR-TB were still not detected in 2014.[1, 6] Treatment scale up lags far behind the Stop TB Partnership's Global Plan targets to treat 1.6 million MDR-TB and XDR-TB patients by 2015, with only 110,803 (23.0%) of estimated incident cases of MDR-TB and 4,044 (8.7%) of estimated incident cases of XDR-TB reported to be enrolled on treatment during 2014.[1] The diagnostic and treatment “gaps” mean that many cases of MDR-TB are neither being identified nor treated, contributing to the further spread of MDR-TB. The consequences of these gaps can be seen in regions reporting increasing rates of MDR-TB among new TB patients.[7] Although evidence from resource-limited settings on the effectiveness and feasibility of PMDT has provided a foundation for guidance, many knowledge gaps still remain.

Two previously published research agendas (2003, 2008) highlighted research gaps affecting the operation of PMDT pilot projects, and identified research questions that needed to be addressed in order to inform the scale up of PMDT.[8, 9] Publications citing the last research agenda spanned all topics in the 2008 publication including Laboratory Support,[10] Epidemiology,[11] Programmatically Relevant Research,[12–15] Treatment Strategy,[16–19] and Management of Contacts,[20–22] as well as those that were cross-cutting through multiple priority areas.[23, 24] These publications included both original research articles and reviews summarizing existing evidence. Here, we update the pending research questions, systematically identify new knowledge gaps, and determine the relative priority of these research questions through a consultative process among research stakeholders in PMDT.

## Methods

### Development of research questions

A group consisting of members from the former Research Subgroup of the MDR-TB Working Group of the Stop-TB Partnership (merged with the Research Task Force of the Global Drug-Resistant TB Initiative [GDI, http://www.stoptb.org/wg/mdrtb/]), Core Group of the GDI, and RESIST-TB (Research Excellence to Stop TB Resistance) (http://www.resisttb.org/) prepared the present manuscript. The group first reviewed the 2008 research agenda on PMDT to classify the previously published research priorities as still relevant and unresolved, relevant and only partially resolved, or no longer relevant.[9] To identify additional knowledge gaps, we reviewed resources including guidelines, documents, websites and publications published between January 2008 and August 2013 from relevant organizations and authorities such as the US Centers for Disease Control and Prevention (CDC), the National Institute of Allergy and Infectious Diseases (NIAID), the WHO, European Centre for Disease Prevention and Control (ECDC), Médecins Sans Frontières (MSF), and others (See Table 1). In addition, we reviewed all PubMed-indexed documents that cited the 2008 publication. We extracted statements about knowledge gaps and/or explicit research questions from these documents. Overlapping statements were consolidated and formulated into 56 research questions that were categorized into 34 subcategories. These were organized into the 5 main research categories, established in the 2008 research agenda on PMDT: Laboratory Support, Epidemiology, Programmatically Relevant Research, Treatment Strategy, and Management of Contacts.[9] From this extensive list of research questions, an online survey to establish priorities among the questions was constructed using Qualtrics (Provo, UT).

**Table 1:** List of documents, websites, and articles citing the 2008 research agenda reviewed for statements on research needs.

CDC Plan to Combat Extensively Drug Resistance Tuberculosis, Recommendations for the Federal Tuberculosis Task Force (2009)
NIAID Research Agenda: Multidrug Resistance and Extensively Drug Resistant Tuberculosis (2007)
MSF/PIH Manual- Tuberculosis: Practical Guide for Clinicians, Nurses, Laboratory Technicians and Medical Auxiliaries (2013)
WHO/Stop TB Operational Research Guide: Priorities in Operational Research to Improve Tuberculosis Care and Control (2011)
The Union: Guidelines for the Clinical and Operational Management of DR-TB (2013)
STOP-TB Partnership Global Plan to Stop TB (2011–2015)
ECDC Technical Report ERLN-TB Expert Opinion on the Use of the Rapid Molecular Assays for the Diagnosis of Tuberculosis and Detection of Drug Resistance (2013)
WHO Guidelines for the Programmatic Management of Drug-Resistance Tuberculosis Emergency Update 2008 (2008)
WHO Guidelines for the Programmatic Management of Drug-Resistance Tuberculosis 2011 Update (2011)
WHO and TDR-Priorities for Tuberculosis Research: A Report of the disease reference group on TB, leprosy and Buruli ulcer (2013)
STOP-TB Partnership: An International Roadmap for Tuberculosis Research (2011)
TB Alliance Website
Stop TB Working Group on New Drugs Website
New Diagnostics Working Group Website
NIH RePORTER
15 Pubmed indexed articles citing the 2008 research agenda [10–24]

### Format and distribution of survey

The survey comprised two primary sections: 1) selection and ranking of specific research questions within each of the five main categories; and 2) global ranking of subcategories. For the first section, respondents were instructed to select 5 priority research questions in each main category identified in the 2008 research agenda on PMDT.[9] Within each main category, they were asked to rank the 5 selected research questions from 1–5, with 1 being the highest priority. Respondents could also choose to select, but not rank research questions. In the second part, respondents were instructed to select and rank 5 priority subcategories from the complete list of subcategories. This second ranking served to assess internal consistency. Additionally, it provided respondents the chance to prioritize not only within a main category but *across* main categories.

Snowball sampling was used to select survey respondents: the survey was distributed by email to PMDT research stakeholders including RESIST-TB, Treatment Action Group, TB CARE I, TB TEAM, Stop TB Partnership’s New Diagnostics Working Group, the former MDR-TB Working Group, and GDI. These groups, in turn, shared the survey with their own distribution lists and with other groups. The number of recipients was estimated to be at least 500.

### Analysis of survey

For each main category, we designated the subset of the survey respondents who selected at least one research question. We calculated the proportion who selected each individual question. Questions that were selected but not ranked were recoded at the intermediate value of 3, and any questions that were unselected by a participant were coded as 6, to reflect their priority as lower than the lowest possible ranking for selected research questions. We then scored each research question as the mean rank, on a scale of 1 to 6, with 1 representing the highest possible priority and 6 the lowest. Because there were different numbers of questions in each category, numeric priority ranking could be compared within, but not between, categories. We performed two different sensitivity analyses to assess the robustness of the rankings. In one, we excluded (rather than recoded) the questions that were not ranked (which comprised <20% of responses). In another, we separately coded unselected questions as missing and weighted the mean by response rate [$Mean\times \frac{\left(N+N_{miss}\right)}{N}$].

For the subcategory ranking, we included responses from individuals who completed this section as instructed, selecting 4, 5, or 6 subcategories from the complete list of 34 subcategories. Selected subcategories were ranked on a scale of 1 to 6, with 1 as the highest priority and 6 as the lowest priority. Subcategories selected but not ranked were recoded to an intermediate value of 3, as for the research questions within the main categories. If a respondent selected or ranked more than 6 subcategories, we excluded the response from the analysis.

### Ethics statement

Ethics approval was not sought for this study. Surveys were completed anonymously and no data (identifiers or otherwise) were collected on respondents.

## Results

**Fig 1:**
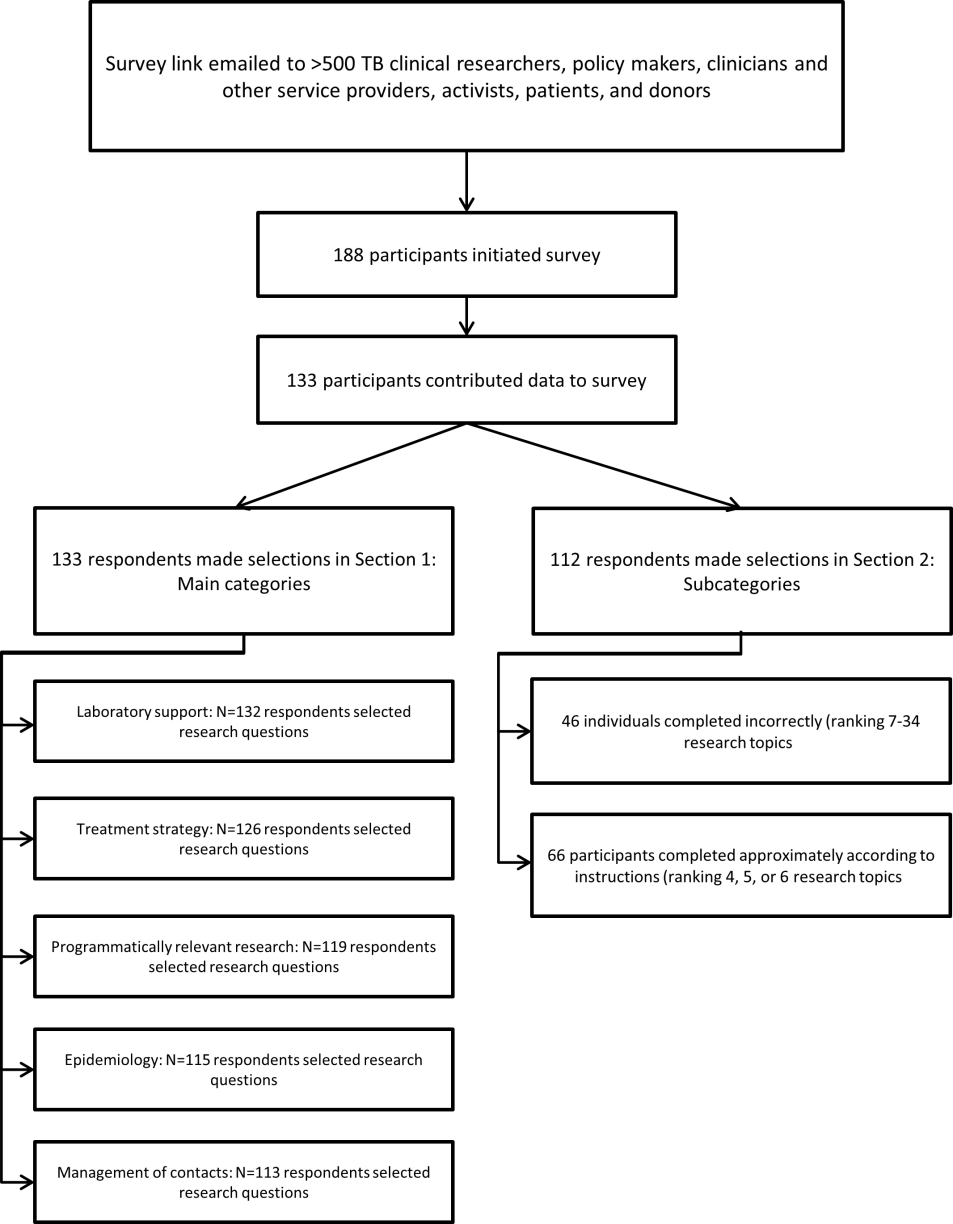
Survey Response.

We identified and reviewed 30 sources for statements on knowledge gaps in PMDT (Table 1): 15 were normative documents or websites; and a PubMed search for peer-reviewed publications that cited the 2008 research agenda returned an additional 15 sources. Over 100 calls for research on topics related to PMDT were extracted from these documents and consolidated into 56 distinct research questions. Since all 5 research categories from the 2008 research agenda were judged to still be relevant, (i.e., none was thought to be completely resolved), the unique research questions were classified across these five categories: 10 in Laboratory Support; 7 in Treatment Strategy; 18 in Programmatically Relevant Research; 13 in Epidemiology; and 8 in Management of Contacts. Survey participants selected priority questions within and across these categories. Among more than 500 survey recipients, 133 (approximately 27%) answered at least one question (Fig 1).

**Fig 2:**
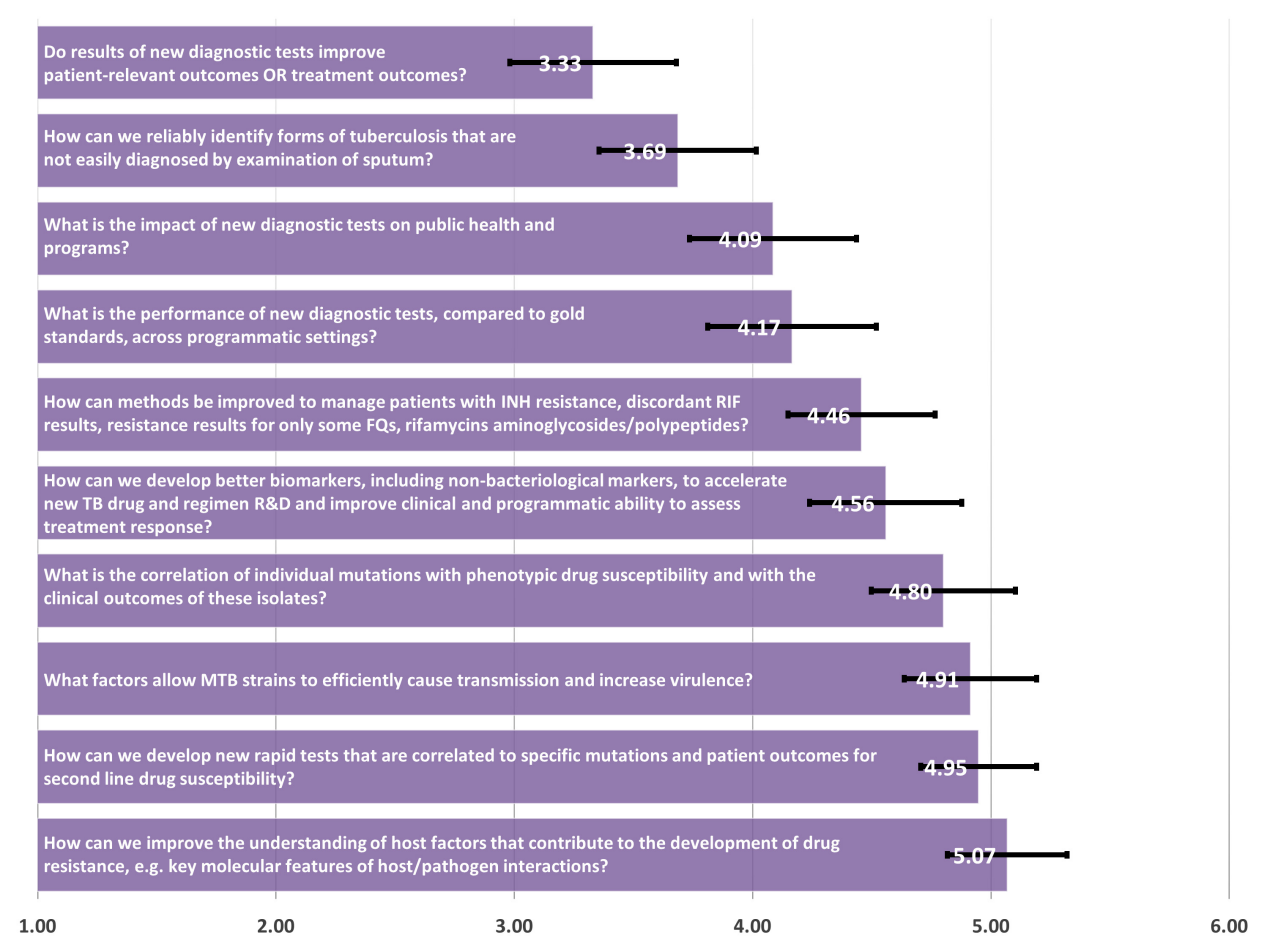
Laboratory support–mean of priority ranking assigned by respondents (N = 132), 1 representing the highest priority and 6 representing the lowest priority.

In the Laboratory Support main category, the questions “Do results of new diagnostic tests improve patient-relevant outcomes OR treatment outcomes?” and “How can we reliably identify forms of tuberculosis that are not easily diagnosed by examination of sputum (e.g., meningitis, pediatric TB, TB in HIV-coinfected persons)?” were selected as top priorities, with scores of 3.33 and 3.69, respectively (Fig 2). These were selected over questions that focused on refining drug susceptibility testing (DST), markers of fitness, and evaluating treatment response.

**Fig 3:**
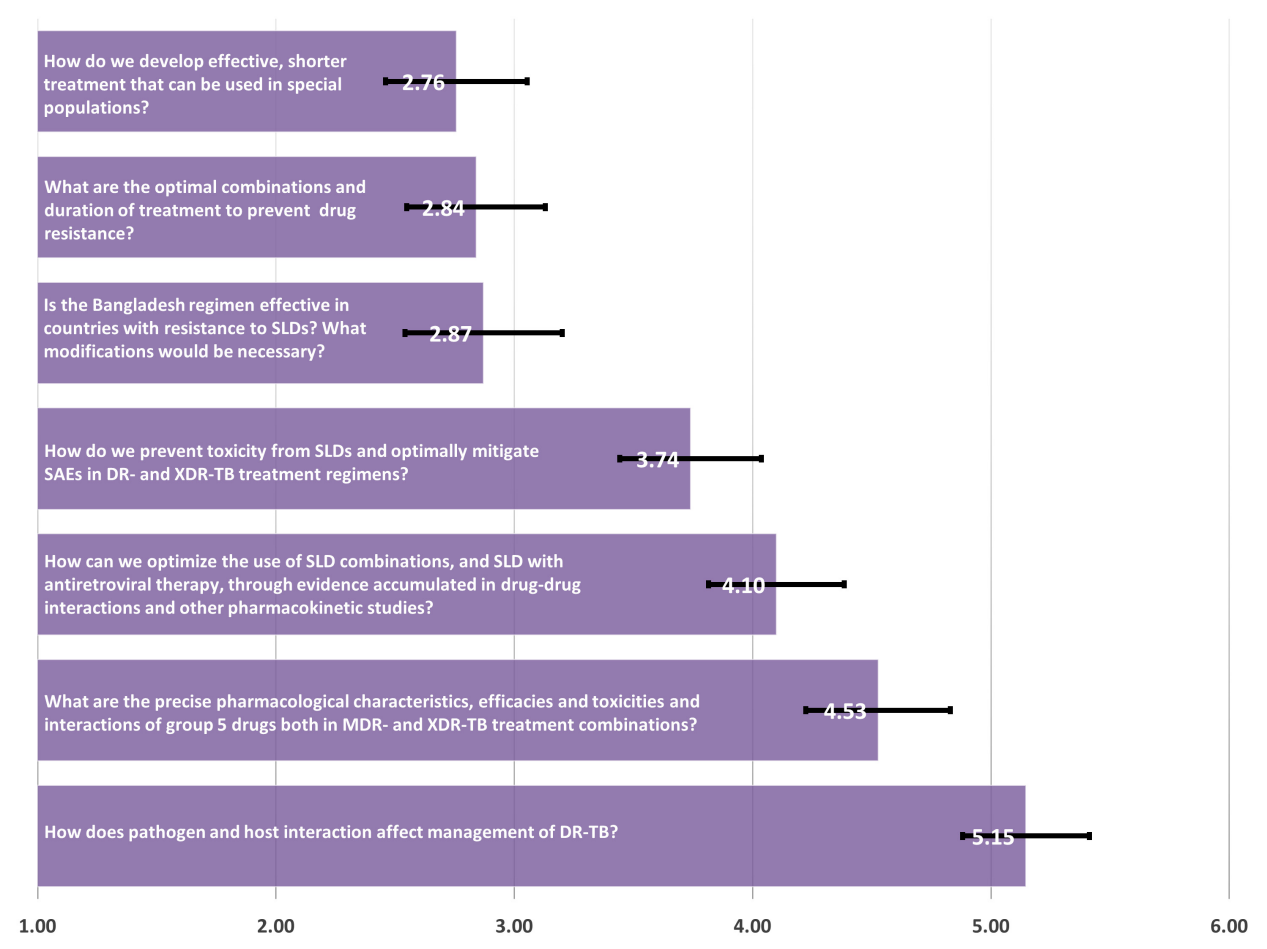
Treatment strategy—mean of priority ranking assigned by respondents (N = 126), 1 representing the highest priority and 6 representing the lowest priority.

In the Treatment Strategy category, the questions: “How do we develop effective and shorter treatment regimens that can be used for special populations (e.g., pregnant/lactating women, children, HIV-coinfected individuals)?”, “What are the optimal combinations and duration of treatment to prevent drug resistance?” and “Is the Bangladesh regimen (a 9-month gatifloxacin-based regimen) effective in countries with high prevalence of resistance to second-line drugs (SLDs)? What modifications would be necessary?” ranked as the top priorities (scores: 2.76, 2.84, 2.87) (Fig 3). The lowest priorities in this category were questions related to optimization and further individualization of treatment with SLDs (with or without antiretroviral therapy) and toxicity.

**Fig 4:**
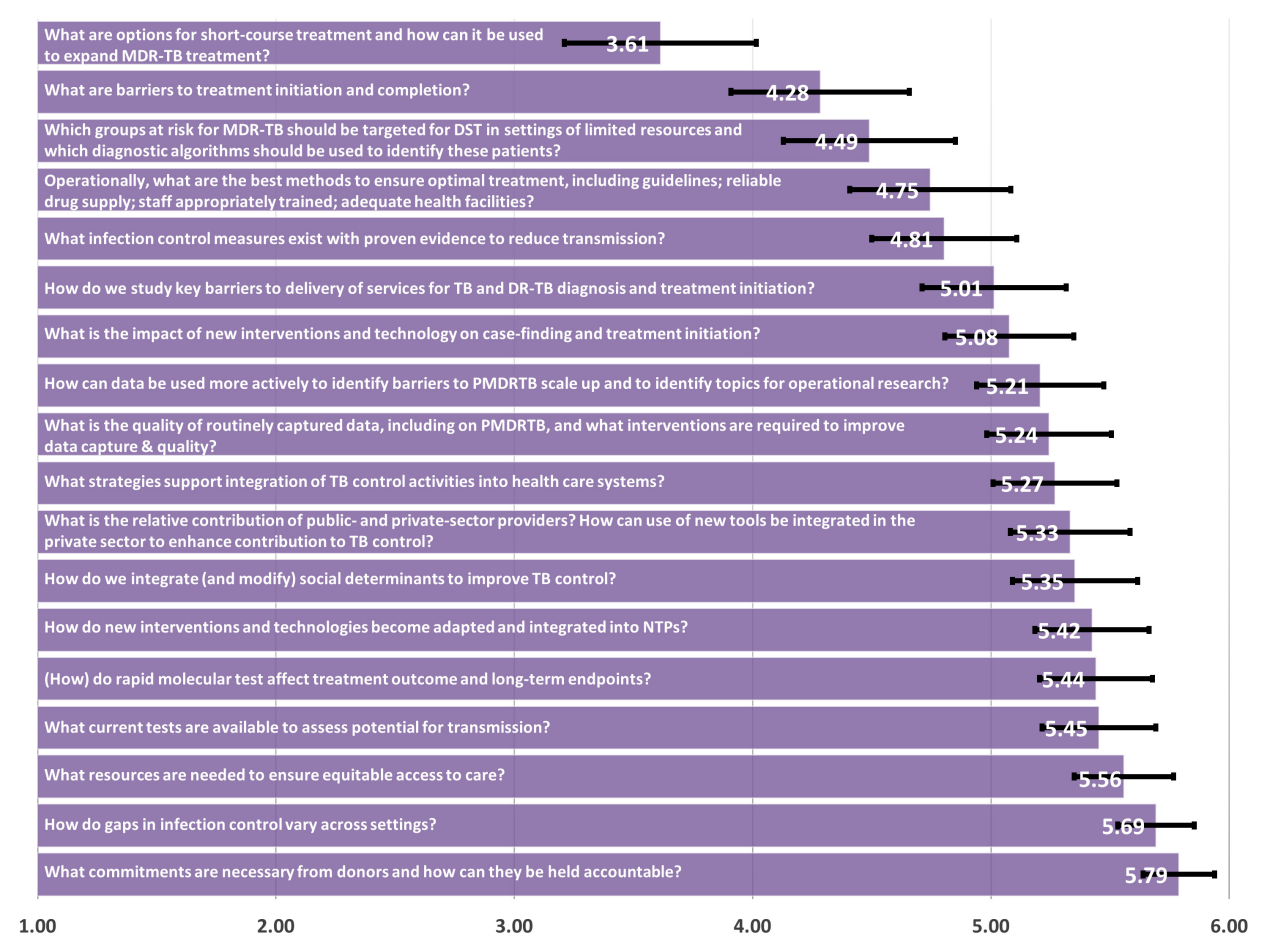
Programmatically relevant research—mean of priority ranking assigned by respondents (N = 119), 1 representing the highest priority and 6 representing the lowest priority.

In the Programmatically Relevant Research category, the highest ranked research question was “What are options for short-course treatment and how can they be used to expand MDR-TB treatment?”. Distant second and third priorities were the questions: “What are barriers to treatment initiation and completion?” (4.28) and “Which groups at risk for MDR-TB should be targeted for DST in settings of limited resources and which diagnostic algorithms should be used to identify patients within risk groups?”(4.49). These were all ranked in the top 5 by those who ranked at least one question, as were “Operationally, what are the best methods to ensure optimal treatment, including guidelines; reliable drug supply; staff appropriately trained; adequate health facilities?” with a score of 4.75 and “What infection control measures exist with proven evidence to reduce transmission?” (4.81) (Fig 4).

**Fig 5:**
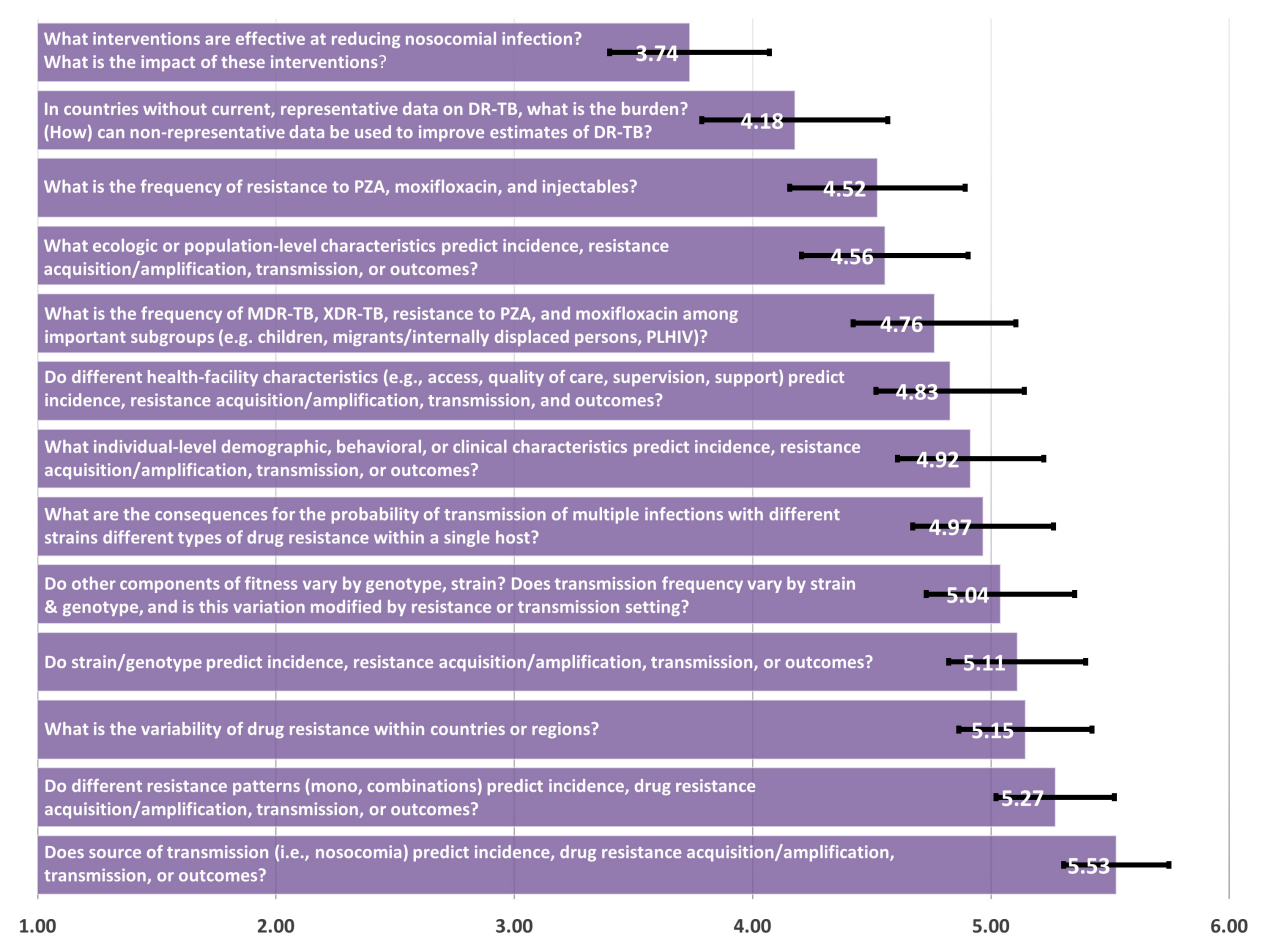
Epidemiology—mean of priority ranking assigned by respondents (N = 115), 1 representing the highest priority and 6 representing the lowest priority.

In the Epidemiology category the question “What interventions are effective at reducing nosocomial infection? What is the impact of these interventions?” was highest ranking, with a score of 3.74. The questions “In countries without current, representative data on the burden of drug-resistant TB, what is the burden? (How) can non-representative data be used to improve estimates of DR-TB?” and “What ecologic or population-level characteristics predict incidence, resistance acquisition and/or amplification, transmission, or outcomes?” were also high ranking, with scores of 4.18 and 4.52, respectively (Fig 5). In addition, the research question “What is the frequency of resistance to PZA, moxifloxacin, and injectables?” also arose as a priority (score: 4.52). The topics with the fewest selections and lowest rankings included the role of strain and resistance patterns as predictors of epidemiologic indicators.

**Fig 6:**
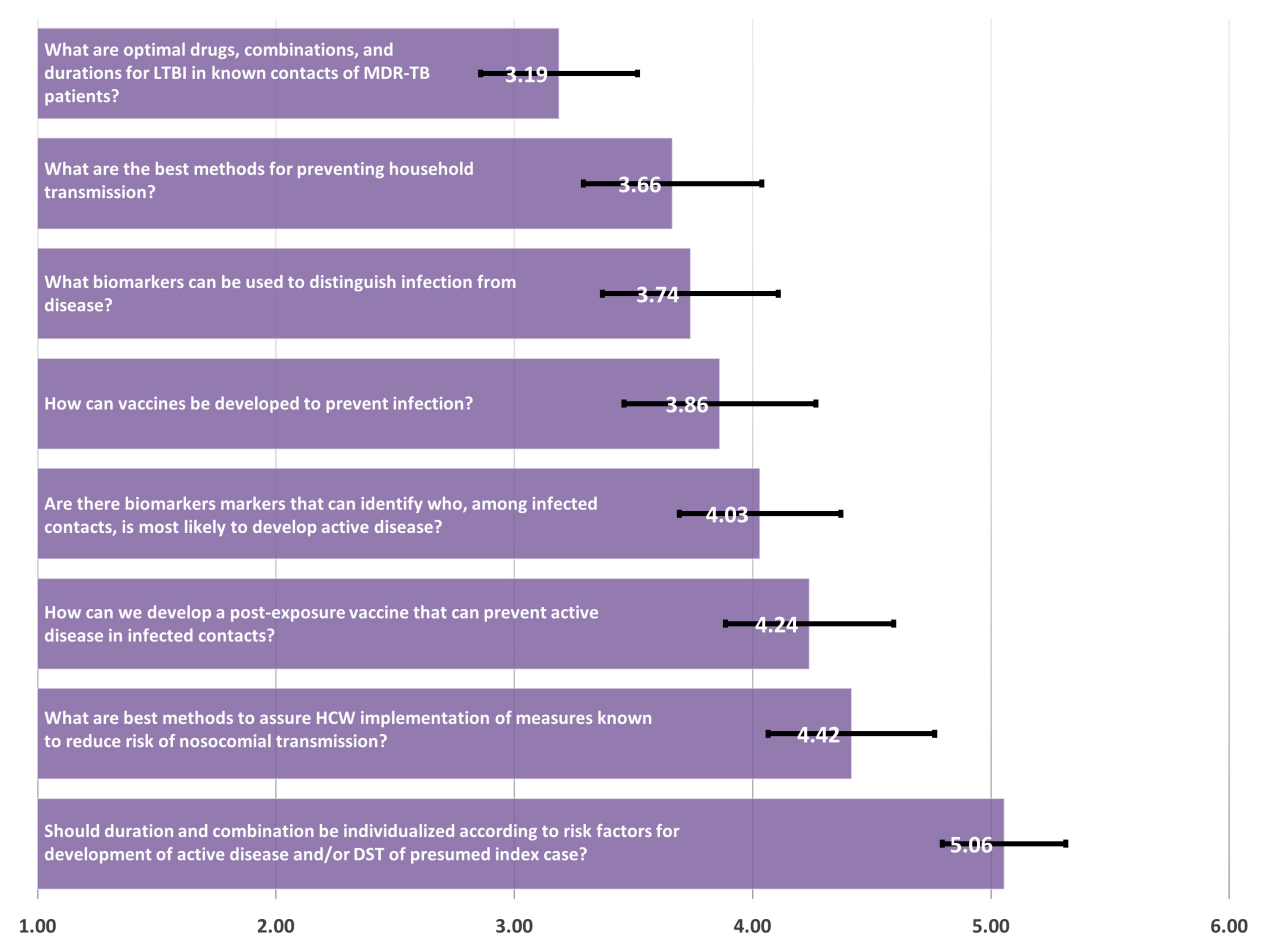
Management of contacts—mean of priority ranking assigned by respondents (N = 113), 1 representing the highest priority and 6 representing the lowest priority.

Lastly, among 113 respondents to the Management of Contacts category, “What are optimal drugs, combinations, and durations for latent tuberculosis infection (LTBI) in known contacts of MDR-TB patients?” was the top priority with a score of 3.17 (Fig 6). Also high priorities were “What are the best methods for preventing household transmission” and “What biomarkers can be used to distinguish infection from disease?” with scores of 3.66 and 3.74, respectively. In contrast, topics on post-exposure vaccines, averting nosocomial transmission, and individualization of prophylactic treatment were lower priorities.

Only 58% of respondents who initiated the survey completed the second part according to instructions (see S1 Appendix). We note, however, that priorities selected across subcategories were generally consistent with those within categories. Results from the Subcategory ranking are available in the Appendix (See S2 Appendix).

Rare exceptions included “vaccines (as a strategy for management of contacts)”, which was selected as the 3^rd^ ranked subcategory overall while the related questions ranked in the middle within the Management of Contacts category. How to optimize access to MDR-TB diagnosis and treatment was selected as 4^th^ in the Programmatically Relevant Research category and 4^th^ overall. Despite these exceptions, the similarity between the rankings of subcategories and main categories supports the internal consistency of survey results. Results from the sensitivity analyses, both when we performed the analysis with results weighted by response rate and when we excluded selections that were not ranked, were similar to those from the primary analysis.

## Discussion

This third research agenda informing PMDT is the result of a multistep, systematic, consultative approach to identify current research priorities. It builds on the pioneering efforts that led to publications in 2003 and 2008.[8, 9] For the present effort, we added an extensive literature review and stakeholder survey to the previous strategy of identifying and grouping priorities by the writing groups.

Certain research questions emerged consistently across categories. Shortened regimens, which would be effective in a range of patient populations, were clearly perceived as a high priority within and across categories. This topic has been the subject of some research in the interim, and the need for advancement in this area was noted in the 2008 research agenda and the 2011 WHO guidelines,[2, 3] the latter calling for further investigation on optimizing treatment regimens, including in special populations such as children. Observational studies of 9-month regimens, variations on that known as the "Bangladesh regimen,” have been successfully implemented in several settings.[25–27] Moreover, the recent conditional approval of bedaquiline and delamanid, two new anti-TB drugs [28, 29], has induced additional interest in shortened, and now all-oral, regimens. Ongoing research efforts to advance the evidence base in this area include the STREAM trial;[26] three trials sponsored by the Global Alliance for TB Drug Development explore shortened regimens for DR-TB: NC-005, ClinicalTrials.gov Identifier: NCT02193776; STAND, ClinicalTrials.gov Identifier: NCT02342886; and NIX-TB, ClinicalTrials.gov Identifier: NCT02333799. Additionally, groups such as the AIDS Clinical Trials Group through ACTG5343, and MSF through TB-PRACTECAL (ClinicalTrials.gov Identifier: NCT02589782) and endTB, together with Partners In Health,[30, 31] are exploring shorter, simpler regimens that depend less on second-line drugs with extensive prior use. Instead, these efforts will use combinations of new drugs or repurposed drugs with less prior population exposure. Interestingly, developing effective shorter regimens—rather than reducing toxicity of treatment—emerged as the priority within the Treatment Strategy category, despite the development of treatment for toxicity linked to second-line drugs appearing as a gap in the 2011 WHO guidance. This may be because shortened regimens with toxicity profiles similar to the current regimen would still be considered an advance if they are effective. Or, this preference may reflect expectations that shorter regimens will reduce exposure to less toxic drugs (e.g., eliminating injectable, shortening exposure to ethionamide).

Treatment of latent *Mycobacterium tuberculosis* infection (LTBI) in contacts of DR-TB patients was also highlighted, as were other measures to prevent or rapidly detect active disease—pre-exposure vaccines, averting nosocomial transmission, and active case finding. Relative to efforts to produce shorter, more efficacious regimens and in contrast to improved LTBI treatment after exposure to drug susceptible TB,[32] there has been limited activity in this domain since the 2008 publication and the 2011 WHO guidelines[3] which also identified this research gap and called for advances in this area. Recent exceptions include case series and observational studies of treatment of LTBI in household contacts of patients with MDR-TB, often children, with regimens containing fluoroquinolones.[33–37] Trials being planned at this writing include V QUIN and TB-CHAMP, which will test levofloxacin for prophylaxis, and PHOENIx (ACTG A5300), which is likely to test delamanid.[38, 39]

Changes from the 2008 research agenda are notable. These include a refinement of the priority placed on new DST methods. In the current research agenda, the call is for better evidence on the impact of new methods on improved treatment outcomes. Research to date has not supported the hypothesis that introduction of rapid molecular tests (GeneXpert MTB/RIF or GenoType MTBDR) is leading to improved treatment outcomes. [40–44] It appears unlikely that diagnostics alone—in the absence of other interventions that improve the timely, programmatic management of MDR-TB—can realistically be expected to have an impact on outcomes or epidemiology of MDR-TB.[42, 45] The current priorities also highlight the need for diagnostic tools that result in improved sensitivity for traditionally difficult-to-diagnose TB (extra pulmonary TB, TB in HIV-coinfected or pediatric populations). The emphasis on programmatic research to select algorithms for screening sub-populations for MDR-TB persists from the 2008 research agenda, but as a relatively low priority in the area of programmatically relevant research. Its decline in perceived importance may be because such risk profiles are already being incorporated in algorithms that guide the use of GeneXpert MTB/RIF and other rapid tests in populations, and, WHO guidelines released in 2011 call for universal screening for rifampin resistance in all TB patients. Working toward this standard obviates the need for refined identification of these risk groups.

Respondents revealed some interest in improved estimates of the prevalence of drug resistance, even in the absence of fully representative national data. This may reflect the reality that even 20 years into the project on Anti-TB Drug Resistance Surveillance, only a limited number of countries have recent, national, representative data from surveys or ongoing surveillance of drug resistance. This is particularly salient for the Africa region in which 19 (40%) of 47 countries have no data and only 28 (60%) countries have or are undertaking national surveys or surveillance. Only 9 (20%) have relatively current data, that is, results from 2010–2014.[1] The absence of recent representative data does not necessarily correspond to the absence of resistance;[46] countries require tools for planning and treating even in the absence of such data.[14]

### Implementation

As noted, the changes in priorities since 2008 reflect, in part, exciting advances in rapid diagnostics—GeneXpert and GenoType MTBDRplus—and treatment options, the Bangladesh regimen and new anti-TB drugs, bedaquiline and delamanid. Some changes also reflect a pragmatism about the current state of affairs—that there would be interest in developing methodologies for drawing inference from non-representative anti-TB drug resistance surveys, for example—and the paucity of resources for TB research.[47] For the same reason, many of the 2008 priorities appear again as priorities eight years later. Also it is important to note that the priorities emerging from these consultative efforts are not necessarily reflected in the choices made by industry. For example, both bedaquiline and delamanid were conditionally approved based on trials whose designs added these drugs to the currently recommended long, weak and toxic standard of DR-TB treatment. This reflects the companies’ priority of getting the drugs approved, and the way regulatory authorities make approval decisions. While short, all-oral regimens emerge here as a top priority, these were not addressed in the sponsors’ trials. Fortunately, non-industry researchers have begun to address these questions, frequently supported by public funding (e.g., South African Medical Research Council, USAID, US National Institutes of Health, UNITAID).[31] Continued advocacy around ensuring adequate resources for research and accelerated testing of new interventions, and support of new initiatives, such as the 3P project, that use open-collaboration frameworks and incentives to promote regimen development early in the clinical development of products is essential.[48] To this end, the engagement of GDI and RESIST-TB, as well as activists, researchers, donors and policymakers will be key to continued research advocacy.

### Limitations

First, the representativeness of survey respondents is not known. The survey sought input from an extensive list of individuals that included clinical researchers, policy makers, clinicians and other service providers, activists, patients, and donors. Only approximately 20% of these individuals responded, and information about geographical and professional distribution of respondents is not available. Nevertheless, since the priorities identified represent progressions—not dramatic deviations—from those identified in the previous documents, the results are credible as a reflection of the perspective of individuals involved in or affected by PMDT. Second, stakeholder input was sought only on issues that were raised in the sources consulted. Although every attempt was made to review an exhaustive body of relevant sources, other knowledge gaps and new areas of important research that were not mentioned in the sources reviewed were not submitted to survey respondents for ranking. This may result in a bias, especially toward applied research, which was prioritized in the previous research agenda on which the current exercise was based. Nevertheless, the systematic, transparent, and inclusive process that resulted in the present document represents a strength of the agenda.

## Conclusion

Longstanding evidence gaps plague the scale-up of programmatic management of MDR-TB. A systematic effort to assess the priorities for evidence necessary to expand treatment access yielded internally consistent, robust results. Coordinated efforts to address questions regarding shorter treatment regimens, distribution of disease where representative data do not exist, and treatment for LTBI in household contacts of known DR-TB patients are essential to stem the epidemic of TB, including DR-TB.

## Supporting Information

S1 Appendix Survey on research priorities in the programmatic management of DR-TB. (PDF)

S2 Appendix Subcategory ranking. (PDF)

S3 Appendix Data. (XLSX)
