# Supplementary material for: Programmatic Management of Drug-Resistant Tuberculosis: An Updated Research Agenda
Source: PLoS One. 2016 May 25;11(5):e0155968. doi: 10.1371/journal.pone.0155968 (PMC4880345; doi:10.1371/journal.pone.0155968)

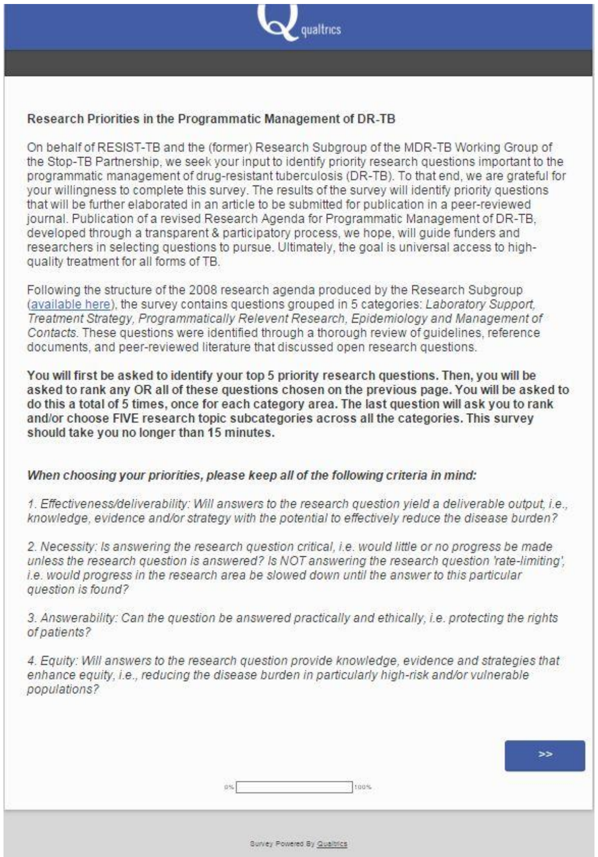

Introduction to the survey

INTRODUCTION

Respondents began the survey with an introduction on the survey purpose, description of the survey's organization, and criteria to assist in guiding prioritization of research questions. Text as it appeared in the survey is below in gray, and a screenshot of the survey introduction page is seen here.

Research Priorities in the Programmatic Management of DR-TB

On behalf of RESIST-TB and the (former) Research Subgroup of the MDR-TB Working Group of the Stop-TB Partnership, we seek your input to identify priority research questions important to the programmatic management of drug-resistant tuberculosis (DR-TB). To that end, we are grateful for your willingness to complete this survey. The results of the survey will identify priority questions that will be further elaborated in an article to be submitted for publication in a peer-reviewed journal. Publication of a revised Research Agenda for Programmatic Management of DR-TB, developed through a transparent & participatory process, we hope, will guide funders and researchers in selecting questions to pursue. Ultimately, the goal is universal access to high-quality treatment for all forms of TB.

Following the structure of the 2008 research agenda produced by the Research Subgroup (available here), the survey contains questions grouped in 5 categories: *Laboratory Support, Treatment Strategy, Programmatically Relevent Research, Epidemiology and Management of Contacts.* These questions were identified through a thorough review of guidelines, reference documents, and peer-reviewed literature that discussed open research questions.

You will first be asked to identify your top 5 priority research questions. Then, you will be asked to rank any OR all of these questions chosen on the previous page. You will be asked to do this a total of 5 times, once for each category area. The last question will ask you to rank and/or choose FIVE research topic subcategories across all the categories. This survey should take you no longer than 15 minutes.

When choosing your priorities, please keep all of the following criteria in mind:

1. Effectiveness/deliverability: Will answers to the research question yield a deliverable output, i.e., knowledge, evidence and/or strategy with the potential to effectively reduce the disease burden?

2. Necessity: Is answering the research question critical, i.e. would little or no progress be made unless the research question is answered? Is NOT answering the research question 'rate-limiting', i.e. would progress in the research area be slowed down until the answer to this particular question is found?

3. Answerability: Can the question be answered practically and ethically, i.e. protecting the rights of patients?

4. Equity: Will answers to the research question provide knowledge, evidence and strategies that enhance equity, i.e., reducing the disease burden in particularly high-risk and/or vulnerable populations?

SELECTION AND RANKING OF RESEARCH QUESTIONS IN MAIN CATEGORIES

Respondents were instructed to first select 5 priority research questions in each main category. Within each main category, they were asked to rank the 5 selected research questions from 1-5, with 1 being the highest priority. Respondents could also choose to select, but not rank research questions. The text provided in the selection and ranking exercise for each main category is below. The Laboratory Support main category is used as an example, though these steps were repeated for each of the 5 main categories.

Laboratory Support

When choosing your priorities, please keep all of the following criteria in mind:

1. Effectiveness/deliverability: Will answers to the research question yield a deliverable output, i.e., knowledge, evidence and/or strategy with the potential to effectively reduce the disease burden?

2. Necessity: Is answering the research question critical, i.e. would little or no progress be made unless the research question is answered? Is NOT answering the research question 'rate-limiting', i.e. would progress in the research area be slowed down until the answer to this particular question is found?

3. Answerability: Can the question be answered practically and ethically, i.e. protecting the rights of patients?

4. Equity: Will answers to the research question provide knowledge, evidence and strategies that enhance equity, i.e., reducing the disease burden in particularly high-risk and/or vulnerable populations?

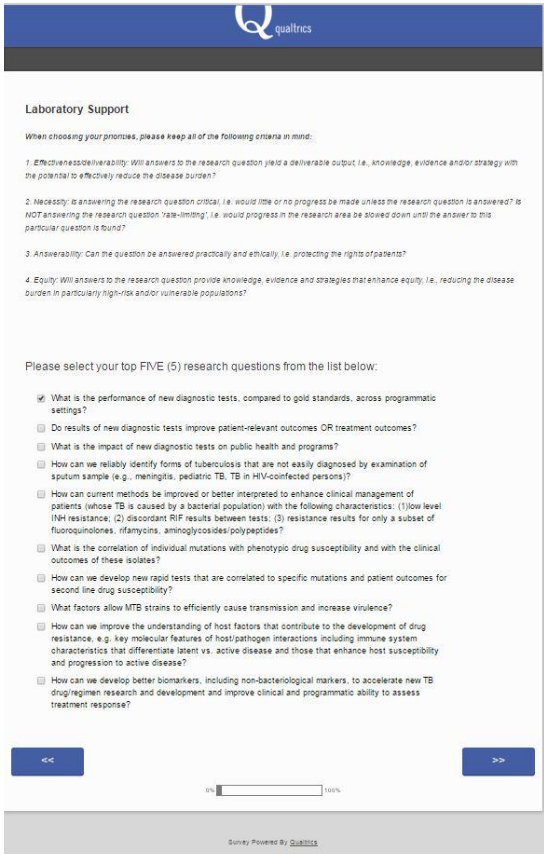

Laboratory Support selection

Please select your top FIVE (5) research questions from the list below:

☐ What is the performance of new diagnostic tests, compared to gold standards, across programmatic settings?

☐ Do results of new diagnostic tests improve patient-relevant outcomes OR treatment outcomes?

☐ What is the impact of new diagnostic tests on public health and programs?

☐ How can we reliably identify forms of tuberculosis that are not easily diagnosed by examination of sputum sample (e.g., meningitis, pediatric TB, TB in HIV-coinfected persons)?

☐ How can current methods be improved or better interpreted to enhance clinical management of patients (whose TB is caused by a bacterial population) with the following characteristics: (1)low level INH resistance; (2) discordant RIF results between tests; (3) resistance results for only a subset of fluoroquinolones, rifamycins, aminoglycosides/polypeptides?

☐ What is the correlation of individual mutations with phenotypic drug susceptibility and with the clinical outcomes of these isolates?

☐ How can we develop new rapid tests that are correlated to specific mutations and patient outcomes for second line drug susceptibility?

☐ What factors allow MTB strains to efficiently cause transmission and increase virulence?

☐ How can we improve the understanding of host factors that contribute to the development of drug resistance, e.g. key molecular features of host/pathogen interactions including immune system characteristics that differentiate latent vs. active disease and those that enhance host susceptibility and progression to active disease?

☐ How can we develop better biomarkers, including non-bacteriological markers, to accelerate new TB drug/regimen research and development and improve clinical and programmatic ability to assess treatment response?

Laboratory Support

If you would like to rank some or all of these research questions, please select a ranking 1-5, 1 being the greatest priority for each research question.

If you would NOT like to rank some or all of these research questions, please select "Unranked."

RESEARCH QUESTIONS FROM PREVIOUS PAGE AUTOPOPULATE BASED ON SELECTIONS

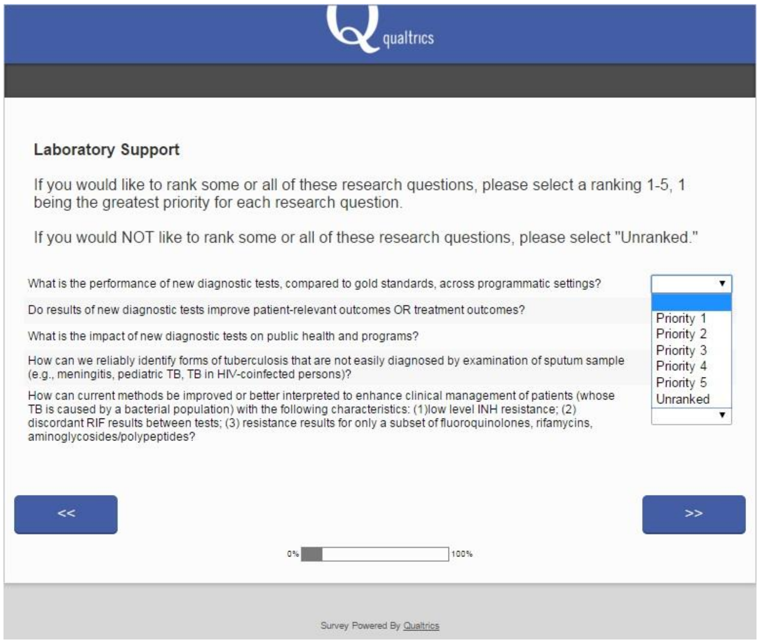

Laboratory Support ranking

The complete list of research questions as they appeared in the survey is listed here:

Laboratory Support

- What is the performance of new diagnostic tests, compared to gold standards, across programmatic settings?
- Do results of new diagnostic tests improve patient-relevant outcomes OR treatment outcomes?
- What is the impact of new diagnostic tests on public health and programs?
- How can we reliably identify forms of tuberculosis that are not easily diagnosed by examination of sputum sample (e.g., meningitis, pediatric TB, TB in HIV-coinfected persons)?
- How can current methods be improved or better interpreted to enhance clinical management of patients (whose TB is caused by a bacterial population) with the following characteristics: (1)low level INH resistance; (2) discordant RIF results between tests; (3) resistance results for only a subset of fluoroquinolones, rifamycins, aminoglycosides/polypeptides?
- What is the correlation of individual mutations with phenotypic drug susceptibility and with the clinical outcomes of these isolates?
- How can we develop new rapid tests that are correlated to specific mutations and patient outcomes for second line drug susceptibility?
- What factors allow MTB strains to efficiently cause transmission and increase virulence?
- How can we improve the understanding of host factors that contribute to the development of drug resistance, e.g. key molecular features of host/pathogen interactions including immune system characteristics that differentiate latent vs. active disease and those that enhance host susceptibility and progression to active disease?
- How can we develop better biomarkers, including non-bacteriological markers, to accelerate new TB drug/regimen research and development and improve clinical and programmatic ability to assess treatment response?

Treatment Strategy

- How do we efficiently develop more effective, shorter and safer M/XDR-TB treatment regimens that may be used for special populations (e.g., pregnant/lactating women, children, HIV-coinfected individuals)?
- What are the precise pharmacological characteristics, efficacies and toxicities and interactions of group 5 drugs both in MDR- and XDR-TB treatment combinations?
- Is the 9 month "Bangladesh" MDR-TB treatment regimen effective in countries with high prevalence of resistance to second-line drugs (SLDs)? What modifications would be necessary?
- How do we prevent toxicity from SLDs and optimally mitigate serious adverse events (SAEs) in DR- and XDR-TB treatment regimens? (especially in special populations e.g., pregnant/lactating women, children, HIV-coinfected individuals)
- How does pathogen and host interaction affect management of DR-TB?
- How can we optimize the use of SLD combinations, and SLD with antiretrovial therapy, through evidence accumulated in drug-drug interactions and other pharmacokinetic studies?
- What are the optimal combinations and duration of treatment to prevent the emergence of anti-TB drug resistance?

Programmatically Relevant Research

- Which groups of patients with increased proportion of MDR-TB should be targeted for DST in the context of limited resources and which diagnostic algorithms should be used to identify patients within risk groups?
- What are options for short-course treatment and how can it be used to expand MDR-TB treatment?
- What are barriers to treatment initiation and completion?
- What infection control measures exist with proven evidence to reduce transmission?
- What current tests are available to assess potential for transmission?
- How do gaps in infection control vary across settings?
- What is the quality of routinely captured data, including on the programmatic management of drug resistant tuberculosis (PMDRTB), and what interventions are required to improve data capture & quality?
- How can data be used more actively to identify barriers to PMDRTB scale up and to identify topics for operational research?
- How do we study key barriers to delivery of services for TB and DR-TB diagnosis and treatment initiation?
- How do new interventions and technologies become adapted and integrated into NTPs?
- What is the impact of new interventions and technology on case-finding and treatment initiation?
- What is relative contribution of public- and private-sector providers? How can use of new tools be integrated, correctly and effectively in private sector, to enhance contribution to TB control?
- What resources are needed to ensure equitable access to care?
- What commitments are necessary from donors and how can they be held accountable?
- (How) do rapid molecular test affect treatment outcome and long-term endpoints?
- What strategies support integration of TB control activities into health care systems?
- How do we integrate (and modify) social determinants to improve TB control?
- Operationally, what are the best methods to ensure optimal treatment, including guidelines; reliable drug supply; staff appropriately trained; adequate health facilities?

Epidemiology

- In countries without current, representative data on the burden of drug-resistant TB, what is the burden? (How) can non-representative data be used to improve estimates of DR-TB?
- What is the frequency of resistance to PZA, moxifloxacin, and injectables?
- What is the variability of drug resistance within countries or regions?
- What is the frequency of MDR-TB, XDR-TB, resistance to PZA, and moxifloxacin among important subgroups: children, migrants/internally displaced persons, people living with HIV?
- Do other components of fitness vary by genotype, strain? Does transmission frequency vary by strain & genotype, and is this variation modified by resistance? Does such strain-dependent transmissibility vary by setting, i.e., hospitals, prisons, mines?
- What are the consequences for the probability of transmission of multiple infections with different strains different types of drug resistance within a single host?
- Do strain/genotype predict incidence? Drug resistance acquisition/amplification? Transmission? And outcomes?

- Do different resistance patterns (mono, combinations) predict incidence? Drug resistance acquisition/amplification? Transmission? And outcomes?
- Do individual-level demographic, behavioral, clinical characteristics predict incidence? Drug resistance acquisition/amplification? Transmission? And outcomes?
- Do different health-facility characteristics (e.g., access, quality of care, supervision, support) predict incidence? Drug resistance acquisition/amplification? Transmission? And outcomes?
- Does source of transmission (i.e., nosocomia) predict incidence? Drug resistance acquisition/amplification? Transmission? And outcomes?
- What ecologic or population-level characteristics predict resistance incidence, acquisition and/or amplification, transmission, outcomes: regimens; population burden of other diseases (e.g., HIV); degree of public-private mix in TB control; geography or socio-economic status?
- What interventions (e.g., household contact tracing; early, routine DST for all TB cases) are effective at reducing nosocomial infection? What is the impact of these interventions?

Management of Contacts

- How can vaccines be developed to prevent infection?
- How can we develop a post-exposure vaccine that can prevent active disease in infected contacts?
- What biomarkers can be used to distinguish infection from disease?
- Are there biomarkers markers that can identify who, among infected contacts, is most likely to develop active disease?
- What are the best methods for preventing household transmission?
- What are best methods to assure HCW implementation of measures known to reduce risk of nosocomial transmission?
- What are optimal drugs, combinations, and durations for LTBI in known contacts of MDR-TB patients?
- Should duration and combination be individualized according to risk factors for development of active disease and/or DST of presumed index case?

RANKING OF SUBCATEGORIES

Respondents were instructed to select and rank 5 priority questions (or subcategories) from the complete list of 43 subcategories. The text provided in the subcategory ranking as it appeared in the survey is below:

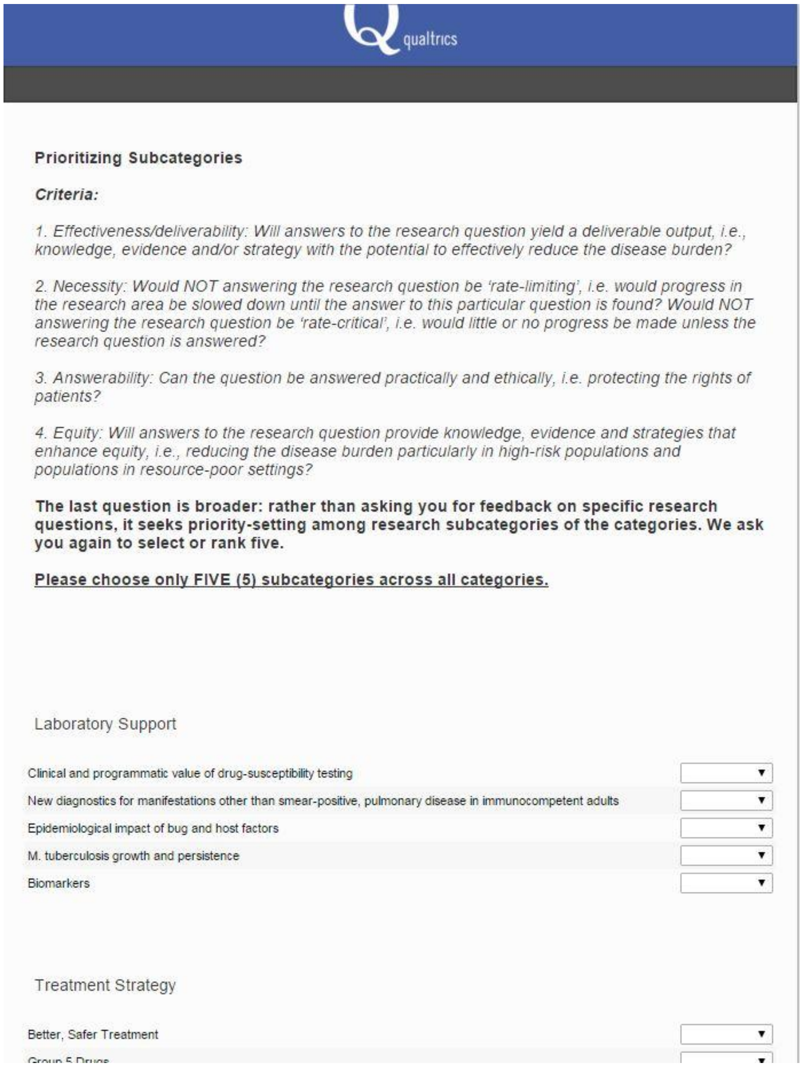

qualtrics

Prioritizing Subcategories

Criteria:

1. Effectiveness/deliverability: Will answers to the research question yield a deliverable output, i.e., knowledge, evidence and/or strategy with the potential to effectively reduce the disease burden?

2. Necessity: Would NOT answering the research question be 'rate-limiting', i.e. would progress in the research area be slowed down until the answer to this particular question is found? Would NOT answering the research question be 'rate-critical', i.e. would little or no progress be made unless the research question is answered?

3. Answerability: Can the question be answered practically and ethically, i.e. protecting the rights of patients?

4. Equity: Will answers to the research question provide knowledge, evidence and strategies that enhance equity, i.e., reducing the disease burden particularly in high-risk populations and populations in resource-poor settings?

The last question is broader: rather than asking you for feedback on specific research questions, it seeks priority-setting among research subcategories of the categories. We ask you again to select or rank five.

Please choose only FIVE (5) subcategories across all categories.

Laboratory Support

Clinical and programmatic value of drug-susceptibility testing

New diagnostics for manifestations other than smear-positive, pulmonary disease in immunocompetent adults

Epidemiological impact of bug and host factors

M. tuberculosis growth and persistence

Biomarkers

Treatment Strategy

Better, Safer Treatment

Subcategory ranking

Prioritizing Subcategories

Criteria:

1. Effectiveness/deliverability: Will answers to the research question yield a deliverable output, i.e., knowledge, evidence and/or strategy with the potential to effectively reduce the disease burden?

2. Necessity: Would NOT answering the research question be 'rate-limiting', i.e. would progress in the research area be slowed down until the answer to this particular question is found? Would NOT answering the research question be 'rate-critical', i.e. would little or no progress be made unless the research question is answered?

3. Answerability: Can the question be answered practically and ethically, i.e. protecting the rights of patients?

4. Equity: Will answers to the research question provide knowledge, evidence and strategies that enhance equity, i.e., reducing the disease burden particularly in high-risk populations and populations in resource-poor settings?

The last question is broader: rather than asking you for feedback on specific research questions, it seeks priority-setting among research subcategories of the categories. We ask you again to select or rank five.

Please choose only FIVE (5) subcategories across all categories.

The complete list of subcategories as they appeared in the survey is listed here:

Laboratory Support
- Clinical and programmatic value of drug-susceptibility testing
- New diagnostics for manifestations other than smear-positive, pulmonary disease in immunocompetent adults
- Epidemiological impact of bug and host factors
- *M. tuberculosis* growth and persistence
- Biomarkers

Treatment Strategy
- Better, safer treatment
- Group 5 drugs
- Standardized, short regimen in settings with prior exposure to second-line drugs
- Toxicity and serious adverse event treatment and management
- Germ and host interaction
- Pharmacokinetics of present second line drugs and drug-drug interactions of second line drugs with ARVs
- Prevention of MDR-TB

Programmatically Relevant Research
- Case finding
- Treatment delivery
- Infection control
- Recording and reporting, use of routine NTP data
- Implementation and dissemination
- Coordination
- Funding and commitment
- Novel methodologies
- Health system strengthening
- Access to MDR-TB: diagnosis, delivery and care management

Epidemiology
- Burden of DR-TB: Geographic areas
- Burden of DR-TB: Subgroups
- Transmission dynamics of DR-TB
- Multi-level risk factors for DR-TB (with regard to incidence, drug resistance acquisition/amplification, transmission and DR-TB treatment outcomes): Organism
- Multi-level risk factors for DR-TB (with regard to incidence, drug resistance acquisition/amplification, transmission and DR-TB treatment outcomes): Person
- Multi-level risk factors for DR-TB (with regard to incidence, drug resistance acquisition/amplification, transmission and DR-TB treatment outcomes): Health facility/services-program
- Multi-level risk factors for DR-TB (with regard to incidence, drug resistance acquisition/amplification, transmission and DR-TB treatment outcomes): Community/Population Level
- Effectiveness and Impact of Interventions

Management of Contacts
- Vaccines
- Preventing spread from source to contact
- Health care training
- Treating LTBI

The survey concluded after the subcategory ranking. The respondent was notified their response had been recorded.

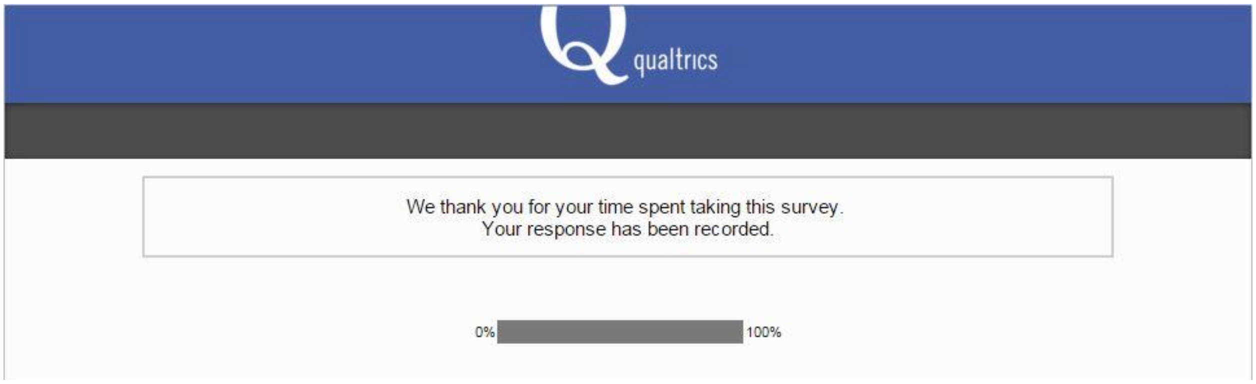

Supplement: S1 Appendix — (PDF) [file pone.0155968.s001.pdf]
