# Supplementary material for: Programmatic Management of Drug-Resistant Tuberculosis: An Updated Research Agenda
Source: PLoS One. 2016 May 25;11(5):e0155968. doi: 10.1371/journal.pone.0155968 (PMC4880345; doi:10.1371/journal.pone.0155968)

S2, Appendix: Subcategories - Mean of Priority Ranking Assigned by Participants (N=66), 1 representing the highest priority and 6 representing the lowest priority

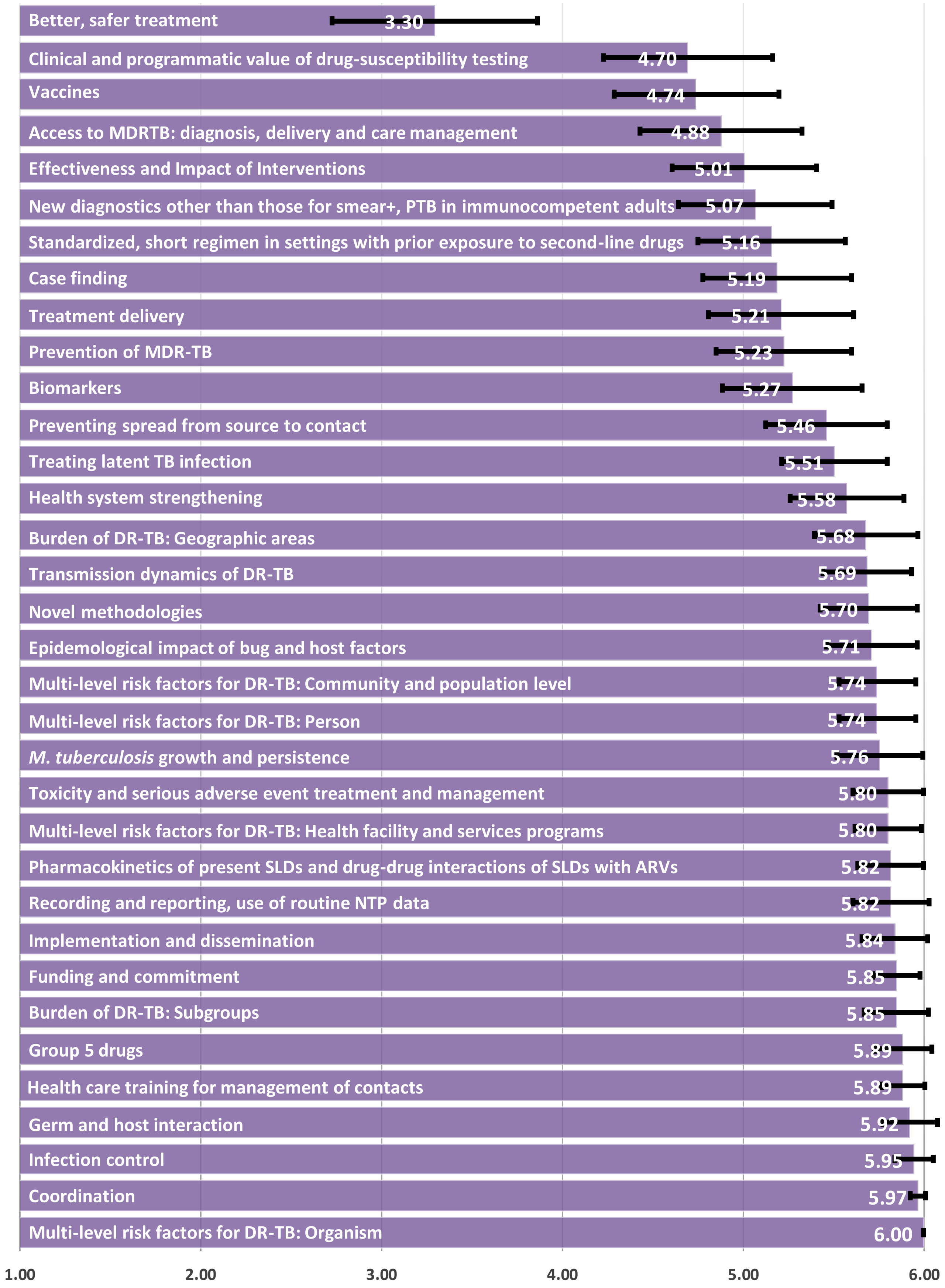

Supplement: S2 Appendix — (PDF) [file pone.0155968.s002.pdf]
